# Ewing's Sarcoma of the Orbit: A Case Report and Literature Review

**DOI:** 10.7759/cureus.48592

**Published:** 2023-11-10

**Authors:** Jahnabi Das, Jyotiman Nath, Chandramouli R, Mostafijur Rahaman, Shiraj Ahmed

**Affiliations:** 1 Department of Radiation Oncology, Dr. Bhubaneswar Borooah Cancer Institute, Guwahati, IND; 2 Department of Radiation Oncology, Krishna Cancer Institute, Cuddalore, IND; 3 Department of Pathology, Dr. Bhubaneswar Borooah Cancer Institute, Guwahati, IND

**Keywords:** ewsr1 translocation, nkx2.2, round cell tumour, orbit, ewing's sarcoma

## Abstract

Ewing's sarcoma (ES) is a malignant small round cell neuroectodermal tumour primarily affecting children in the first and second decade of life.

Since ES is difficult to control, early diagnosis is crucial, and the treatment requires a multimodality approach constituting chemotherapy, surgery and radiotherapy.

Here, we present a case of ES of orbit in a 17-year-old female diagnosed in 2021. This case report focuses on the clinicopathological presentation and management principles of this rare manifestation of the disease that unilaterally infiltrated into the extraconal space of the orbit as well as the I/L frontal sinus. The patient underwent chemotherapy and radiation, and she is on follow-up.

## Introduction

Ewing's sarcoma (ES) is a malignant small round cell neuroectodermal tumour which primarily affects children in the first and second decade of life, classically involving long bones of lower limbs, ribs and the pelvis [1]. Primary Ewing's tumour in the head and neck region is unusual, typically occurring in the mandible and maxillary bone [2]. Occurrence in orbit is rare; these represent metastatic disease from distant sites in most cases. Primary orbital ES was first reported in 1950 [3]. It is the second most common malignant skeletal tumour in children and adolescents, starting in the soft tissues. It accounts for 10% of all bone and 4% of head and neck tumours, with the mandible, maxilla, and skull being the most commonly affected. It is uncommon to have orbital involvement [4].

This tumour has similar morphological aspects of tiny round blue cell tumours, including membrane CD99 protein expression, cytogenetic features of t(11;22) translocation or t(21;22) rearrangement, and molecular features of hybrid transcripts of the EWS gene with FL1 or ERG gene [5]. It is a tumour that follows a highly aggressive course. Primary orbital ES is extremely rare, with just a few individual case reports and case series published in the literature. We present a case of primary orbital ES in a 17-year-old girl with unilateral proptosis of the left eye.

## Case presentation

**Figure 1:**
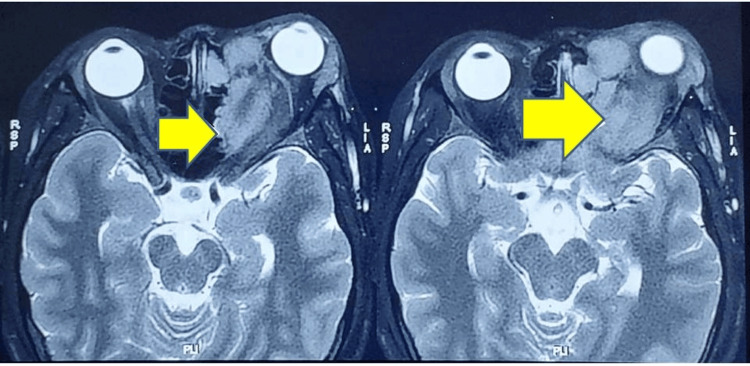
Prechemotherapy T2W MRI images with a yellow arrow pointing at the 4.6x3.9x3 cm sized mass lesion in the left superior and medial orbit compressing the left optic nerve and erosion of the medial wall and the roof of the left orbit. MRI: Magnetic resonance imaging

A 17-year-old girl presented to a local hospital with swelling around the left eye, watering of the eye and blurred vision for three months. MRI showed an extraconal lesion in the left superior and medial orbit, up to the apex, infiltrating the superior rectus, superior oblique and middle rectus muscle approximately 4.6 x 3.9 x 3 cm, with compression of the optic nerve and erosion of the medial wall and the roof of the left orbit (Figure 1).

**Figure 2:**
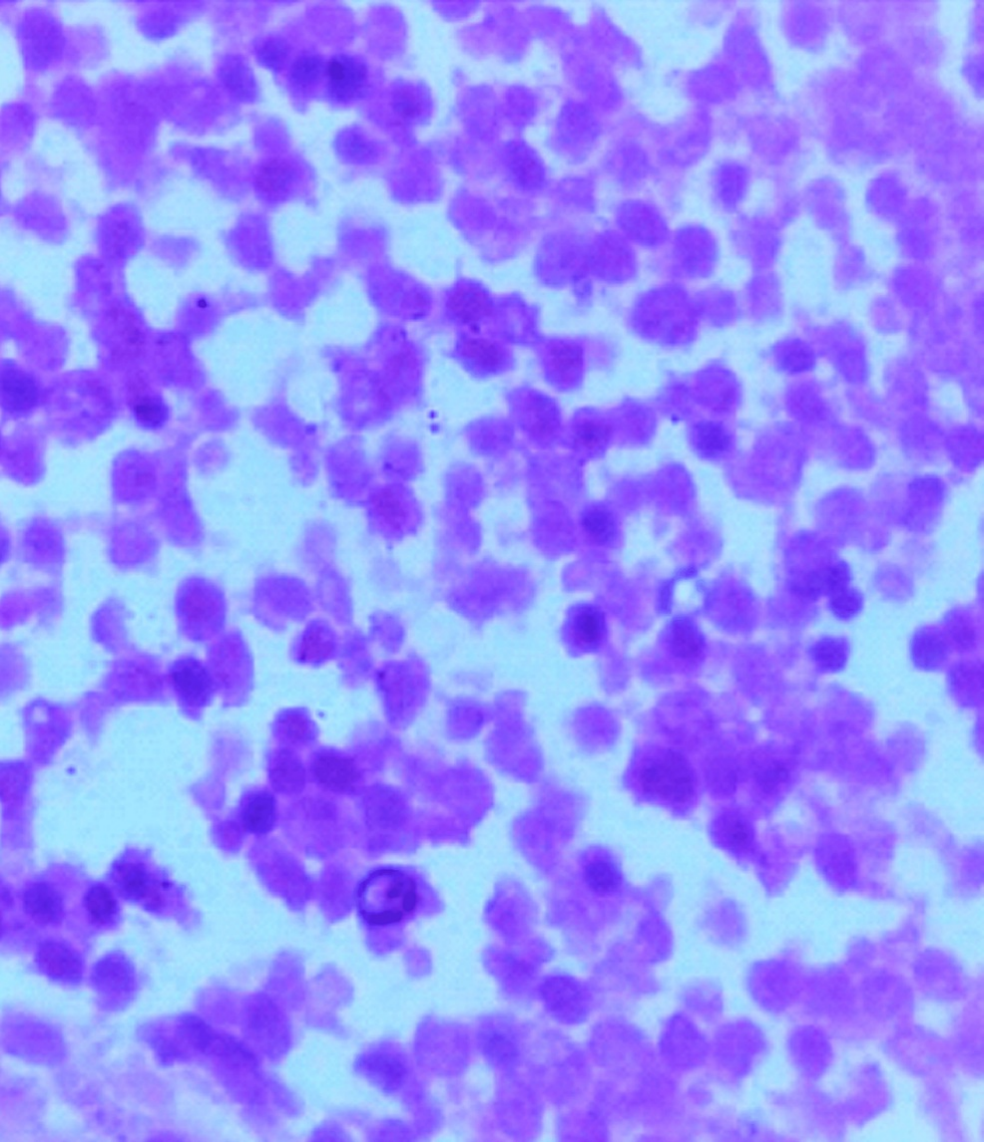
High power view (H&E 40X) showing atypical round cells with hyperchromatic nuclei, inconspicuous nucleoli and scant cytoplasm. H&E: Haematoxylin and eosin

The PET-CT scan revealed a similar finding with SUVmax of 7.5 without a distant tumour spread, so we considered this a primary orbital tumour. Anterior orbitotomy and transnasal endoscopic biopsy were done in a local tertiary eye hospital. The histopathological picture suggested a blue round cell tumour (Figure 2).

Immunohistochemistry was positive for vimentin, CD99 and NKX2.2, with the Ki67 being 80%. Fluorescence In Situ Hybridization (FISH) done for EWSR1 translocation (11, 22) was positive, confirming the diagnosis of ES. Bone marrow aspirate showed reactive marrow, and bone marrow biopsy showed no evidence of malignancy. She was then referred to our hospital for further management.

The institutional multidisciplinary tumour board decided to treat her further with systemic chemotherapy followed by local radiotherapy. Chemotherapy was started as per the Ewing Family Tumor-EFT 2001 protocol. At week 11 of chemotherapy, the patient was started on local treatment with radiation. A contrast-enhanced MRI of the orbit done before the start of radiation showed an infiltrating lesion in the left orbit involving the superomedial compartment, left frontal sinus and left lateral ethmoidal air cells. The necrotic component (25 x 27mm) was seen along the inferior wall of the left frontal sinus. Posteriorly, on the left side, there was a loss of interface with the optic nerve. The contralateral right eye and right optic nerve were normal.

**Figure 3:**
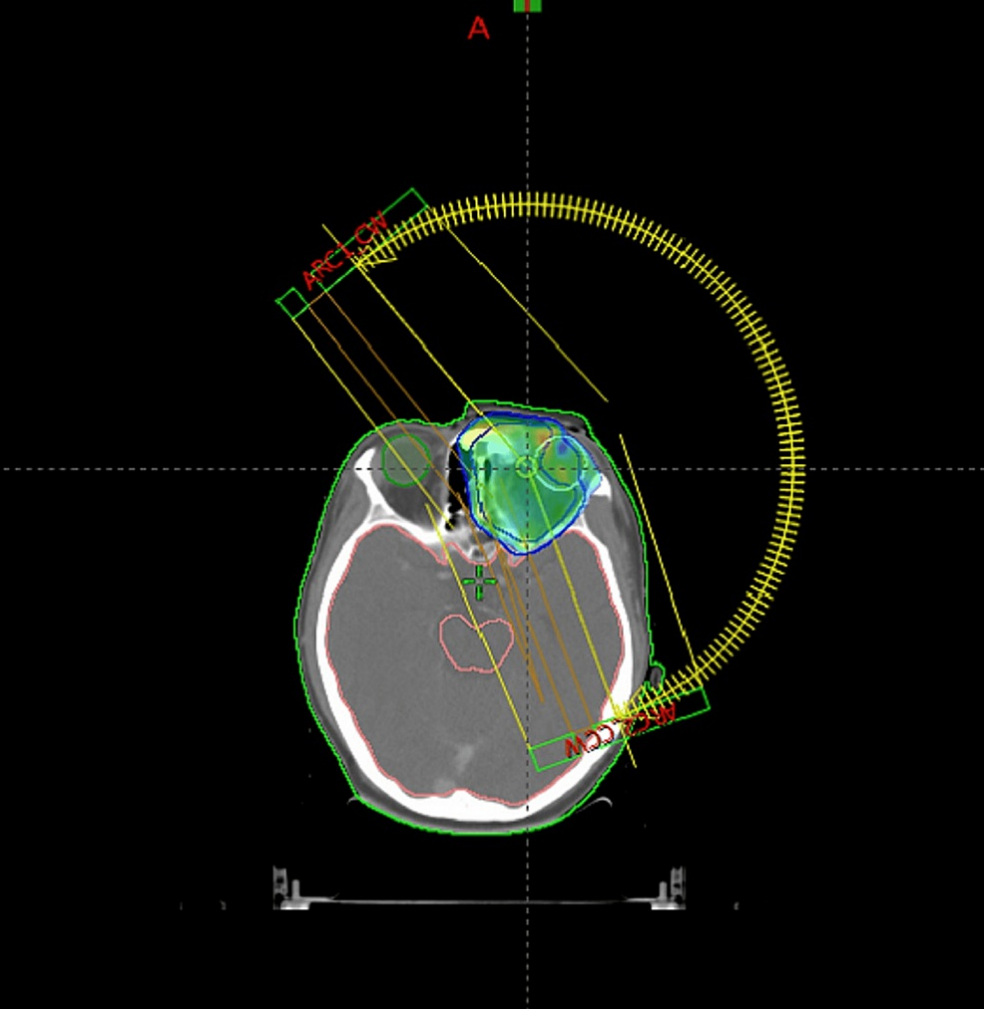
Dose colour wash of 95% prescribed dose in Rapid Arc technique

Baseline visual acuity and perimetry were within normal limits. The patient was immobilised in a supine position with a head and neck thermoplastic mould, and a CT simulation was done with a 3 mm slice thickness. A custom wax bolus of 5 mm was used over the target area during the simulation and treatment. The simulation CT was fused with the pre-chemotherapy MRI, and the gross pre-chemotherapy disease was contoured as gross tumour volume (GTV). The clinical target volume (CTV) comprised the residual tumour with the entire left orbit limited by the normal anatomical barriers. The CTV was given a margin of 5 mm to create the planning target volume (PTV). Radiotherapy was planned using 06 MV photons by Volumetric Modulated Arc Technique-RapidArc technique consisting of two partial arcs in the Eclipse treatment planning system (Varian Medical Systems, Inc. Palo Alto, CA, USA) and the prescription dose was 54 Gy in 30 fractions to the PTV. A total of 95% of the PTV volume received 99.3% of the prescribed dose, i.e. V95 was 99.3% (Figure 3).

The dose to the organs at risk was within the standard tolerance limits, with the mean dose received by the contralateral eye and lens being 14Gy and 6Gy, respectively. The maximum dose received by the optic chiasm, left optic nerve and right optic nerve are 52 Gy, 55 Gy and 25 Gy respectively. Treatment was delivered with a Varian Trilogy linear accelerator (Varian Medical Systems, Inc. Palo Alto, CA, USA). She tolerated radiation well, developing only grade 1 dermatitis (Radiation Therapy Oncology Group-RTOG Acute Radiation Morbidity) during treatment. However, after the completion of treatment, the patient did not turn up for a follow-up visit but on a telephone interview, she informed that she is doing her normal day-to-day activities and attending her regular college classes without difficulty.

## Discussion

A female patient of 17 years with primary ES of the orbit was disease-free with no evidence of disease at the last follow-up, highlighting the reasonably good outcomes expected with multimodality management that included surgery, chemotherapy, and radiation therapy. A previous report by Koka et al. reported the mean age at presentation to be 17.50 years [6]. So far, ES has been reported to occur rarely, only in adults aged 40 to 60 years [7].

In our case, the patient presented with unilateral proptosis, swelling around the eye, watering of the left eye and gradually progressive loss of vision. Woodruff et al. reported about a six-year-old boy whose sole complaints were headache and profound loss of vision, which was acute in onset due to a tumour in the orbit and ethmoid sinus that had spread into the anterior and middle cranial fossa and caused optic nerve injury [8]. The ES of the orbit is usually unilateral, with just a few cases of bilateral ES of the orbit reported in the literature [9].

The osseous component of orbital ES is more common (85%) than the extraosseous (15%) component. Osseous ES has a bony origin and can spread into soft tissue spaces, while the extraosseous ES arises from soft tissue in the orbit [10]. In our study, there is erosion of the medial wall and the roof of the left orbit, with the lesion compressing the left optic nerve.

On CT imaging, ES appears as an infiltrative lytic lesion causing severe bone damage and a mottled look of the bone.

They present heterogeneous masses on MRI that are isointense to hypointense on T1-weighted images, isointense to hyperintense on T2-weighted imaging, and exhibit contrast enhancement with gadolinium [11]. In our study, there were no distant sites of tumour metastasis.

When diagnosing a histopathological picture of a small round blue cell tumour, the differentials will be rhabdomyosarcoma, neuroblastoma, lymphoma and osteogenic sarcoma [12].

ES is a unique neoplasm that arises from undifferentiated small round cells in the bone. This tumour represents 8-10% of all primary malignant bone tumours. Patients under 30 account for 90% of cases, while those under 20 account for 75%. Its frequency is highest in 5 to 13 years of age [13].

ES has an unclear histogenesis. Because vascular pathways were frequently present, Ewing hypothesized that this tumour had a vascular origin and called it endothelial myeloma of bone [14]. Most cases of ES in the head and neck area are seen in the maxilla and mandible. Orbital ESs are more commonly secondaries rather than the primary [15].

Extraskeletal sarcomas are clinically and pathologically indistinguishable from bony ES and have been described most commonly in young adults. They may degrade neighbouring bone, making the precise location of origin challenging to establish. Extraosseous tumours are more uncommon than bone tumours, and they may represent numerous histologic tumour types rather than a single entity. Although these lesions have prominent amounts of cytoplasmic glycogen and poorly formed cell junctions, electron microscopy, immunohistochemistry, and chromosomal analyses suggest that at least some of these lesions are other tumours, such as peripheral neuroepithelioma or other primitive soft-tissue tumours [16,17].

Until recently, the outlook for ES at all sites was similarly bleak. The five-year survival rates with surgery and radiation alone were only 8% and 10%, respectively [6]. According to Boyer et al., there is a 58% mortality rate from metastatic disease in the first year following diagnosis and an overall mortality rate of 88% after 4-10 years of treatment with surgery, radiotherapy, or both. They hypothesized this was due to subclinical metastasis at the presentation time rather than local therapy failure. With the addition of adjuvant treatment, the prognosis has significantly improved, with five-year survival rates increasing to 53%. Local recurrence has also been reduced from 38% following radiotherapy alone to 5% following adjuvant chemotherapy. When symptoms improve, the prognosis appears to improve [18]. The following table shows the summary of a few reported cases (Table 1).

**Table 1:** Review of literature on primary Ewing's sarcoma involving the orbit. NS = not specified; RT = radiotherapy; CT = chemotherapy; NACT = neoadjuvant chemotherapy; NA = not available; FNAC = fine needle aspiration cytology; M = male; F = female

S. No.	Study	Year	No. of cases	Age (years)	Sex	Treatment	Status at last follow-up	Follow-up
1	Harbert and Tabor et al. [3]	1950	2	19	M	RT=30 Gy	Alive	Ten months
				24			Died	
2	Alvarez-Berdecia et al. [19]	1979	1	6	M	Exision+RT(NS)+CT	Alive	Seven months
3	Woodruff et al. [8]	1988	1	6	M	Biopsy+RT(50 Gy)+CT	Alive	Nine months
4	Lam et al. [9]	1999	1	2	M	Biopsy+CT+RT(45 Gy)	Alive	30 months
5	Dutton et al. [15]	2000	2	2.5	M	NA	Died	17 months
				7				NA
6	Kaliki et al. [20]	2017	9	0.4-28	2M; 7F	NACT+EXCISION BIOPSY/DEBULKING+RT	Eight died	21 months
7	Koka et al. [6]	2021	8	14	7M; 1F	Chemo+Surgery+RT	one died; seven alive	Average 41 months
8	Present study	2021	1	15	1F	Biopsy+Chemo+RT	alive	12 months

## Conclusions

In summary, ES is a bone tumour that seldom affects the orbit. Most youngsters, with a small male predominance, are affected in their second decade. The instances manifest with distinct symptoms, and local trauma may predispose to the diagnosis of an asymptomatic cancer that was previously present. Neuroblastoma, rhabdomyosarcoma, and lymphoma are all possible diagnoses. Histopathological and immunohistochemical studies are used to confirm the diagnosis. Follow-up for cases with orbital involvement is insufficient in the current literature. Local excision or radiotherapy, as well as systemic chemotherapy, is used to treat the condition.
